# Collagen wrapping and local platelet-rich fibrin do not improve the survival rates of ACL repair with dynamic intraligamentary stabilization: a retrospective case series after ≥5 years postoperatively

**DOI:** 10.1186/s40634-022-00517-4

**Published:** 2022-08-08

**Authors:** Sophie C. Eberlein, Vanessa Rodriguez, Andreas Hecker, Katharina Schürholz, Sufian S. Ahmad, Frank M. Klenke

**Affiliations:** 1 Department of Orthopaedic Surgery and Traumatology, Inselspital, Bern University Hospital, University of Bern, Freiburgstrasse 4, 3010 Bern, Switzerland; 2 Department of Orthopaedic Surgery and Traumatology, Kantonsspital Aarau, Aarau, Switzerland; 3 Orthopaedic Department of the Medical School of Hannover, Annastift Hospital, Hannover, Germany

**Keywords:** Anterior cruciate ligament, Primary anterior cruciate ligament repair, Dynamic intraligamentary stabilization, Ligamys

## Abstract

**Purpose:**

Anterior cruciate ligament (ACL) repair has been recommended as a treatment principle for ACL tears. Several authors have advocated a potential role for primary repair techniques in the ACL decision tree. However, long-term results have been controversial. This study aims to determine the survival of the primarily repaired ACL after dynamic intraligamentary stabilization (DIS) with and without augmentation.

**Methods:**

Between 2014 and 2019, 102 patients with isolated proximal ACL ruptures underwent DIS repair within 21 days from injury and were available for follow-up either clinically or telephonically after ≥5 years postoperatively. In 45 cases, DIS repair was augmented with collagen fleece wrapping, platelet-rich fibrin (PRF) or both. Failure was defined as traumatic re-rupture or conversion to ACL reconstruction. The patients being available for physical examination underwent a.-p. stability measurement with a KT-1000 device. Functional outcome was measured with the IKDC, Tegner and Lysholm scores. Kaplan-Meier survival analysis, Log-Rank Test and Binominal logistic regression were performed.

**Results:**

After a minimum 5-year follow-up, 71/102 (69.6%) DIS repairs were not re-reptured and clinically and/or subjectively stable. Augmentation did not improve survival rates (*p* = 0.812). The identified factors influencing failure were a younger age and a pre-injury Tegner activity level of ≥7. 95.7% of those patients with an intact ACL repair had normal or near normal knee function based on the IKDC scoring system.

**Conclusions:**

The 5-year overall survival rate of DIS was 69.6%. Collagen fleece wrapping and local PRF application did not improve survival. Patients not suffering failure of repair demonstrated high satisfaction. Nevertheless, the results are inferior to those of established ACL reconstruction procedures.

**Level of evidence:**

Case series, Level IV.

## Background

Anterior cruciate ligament (ACL) tears are the most common ligamentous injuries in the human knee. The first aim of ACL repair is to restore knee stability and reduce pain. The second is to avoid long-term complications, like osteoarthritis [28].

Over the past decades non-surgical and surgical techniques of the ruptured ACL have been described [10, 14, 27]. The most common recommendation for complete ACL tears in a young and active population is the reconstruction with an autologous tendon graft [4, 26]. One disadvantage of anterior cruciate ligament reconstruction (ACLR) is donor-site morbidity when using autologous tendon grafts [11]. Moreover, ACLR includes arthroscopic debridement of the native ACL tissue, which consists of a fan-shaped bundle of 17 different ligament fascicles.- The autologous tendon grafts used in ACLR are not able to reconstitute the exact rotational kinematics of the uninjured knee [15, 24] .The existence of a reflex from the afferent nerves of the cruciate ligaments to the muscles around the knee has been demonstrated, consisting of mechanoreceptors, which are mainly located near the femoral and tibial attachments of the ACL [22, 30]. The loss of proprioception might be one reason for the high rates of posttraumatic osteoarthritis that cannot be prevented by reconstruction [16, 29].

Dynamic intraligamentary stabilisation (DIS) using Ligamys (Mathys Ltd., Bettlach, Switzerland) was introduced and first used in 2009 after being successfully tested in human cadaver and sheep model [17, 18]. Since then, several authors have advocated a potential role for DIS in the ACL decision tree [1, 6, 21].

However, mid- and long-term results have been controversial. Henle et al. presented excellent results in a case series in short- to mid-term follow-up intervals with failure rates as low as 4% [13]. However, higher failure rates of the technique ranging from 15% to 30% have been described after mid- to long-term follow-up [1, 3, 7, 21, 23].

An increased cell proliferation of anterior cruciate ligamentocytes treated with platelet-rich plasma (PRP) has been observed in-vitro by Krismer et al. [20]. Another in-vitro study on osteoblasts has shown that PRF application led to more controllable and long-term release of growth factors like transforming growth factor (TGF)**-**β1 and platelet-derived growth factor (PDGF)-AB compared to PRP [12]. PRF may facilitate the release of growth factors at the application site over a period of several days, as this has been shown for Choukroun’s PRF [5].

As primary repair of ACL ruptures with intact synovial coverage has shown to have higher healing capacities [3], we hypothesized that additional augmentation of the primary repair may improve the healing rate of the ACL after DIS repair. To this end, a new augmentation technique with circular collagen matrix wrapping and PRF attachment at the rupture site was established to simulate the synovial coverage of the ACL. Evangelopoulos et al. reported less complication rates of DIS, including extension deficit and re-rupture if a collage I/III membrane was additionally applied to the mid-substance ACL repair [8].

The aim of this study was to report the survival after DIS of isolated proximal ACL ruptures with and without augmentation using a collagen fleece wrapping and/or local PRF.

## Methods

Between July 2009 and February 2014, 127 patients with isolated ACL ruptures underwent DIS repair within median 11 days (minimum 0, maximum 21 days) from injury at an academic institution (single-center).

Inclusion criteria were defined as: > 18 years of age, closed growth plates, diagnosed with an isolated proximal ACL rupture based on MRI and in intraoperative findings, performance of surgery within 21 days from the day of injury and a minimum postoperative period of 60 months.

Exclusion criteria were multi-ligamentous injuries (MCL tears grade 1 were not excluded) and additional injuries to the same leg, like fractures.

**Fig. 1:**
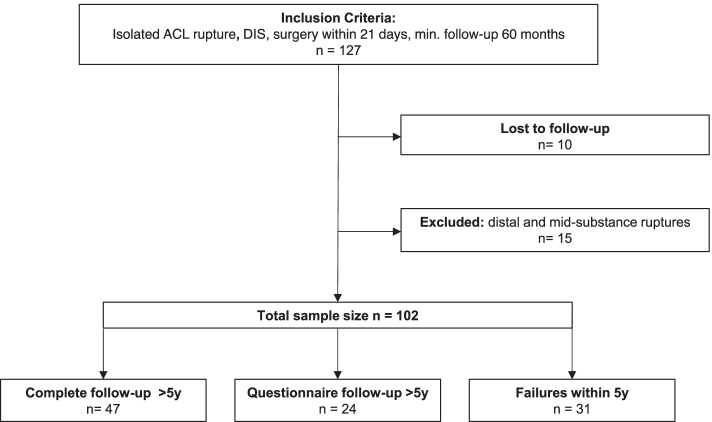
Flowchart illustrating patient inclusion

**Fig. 2:**
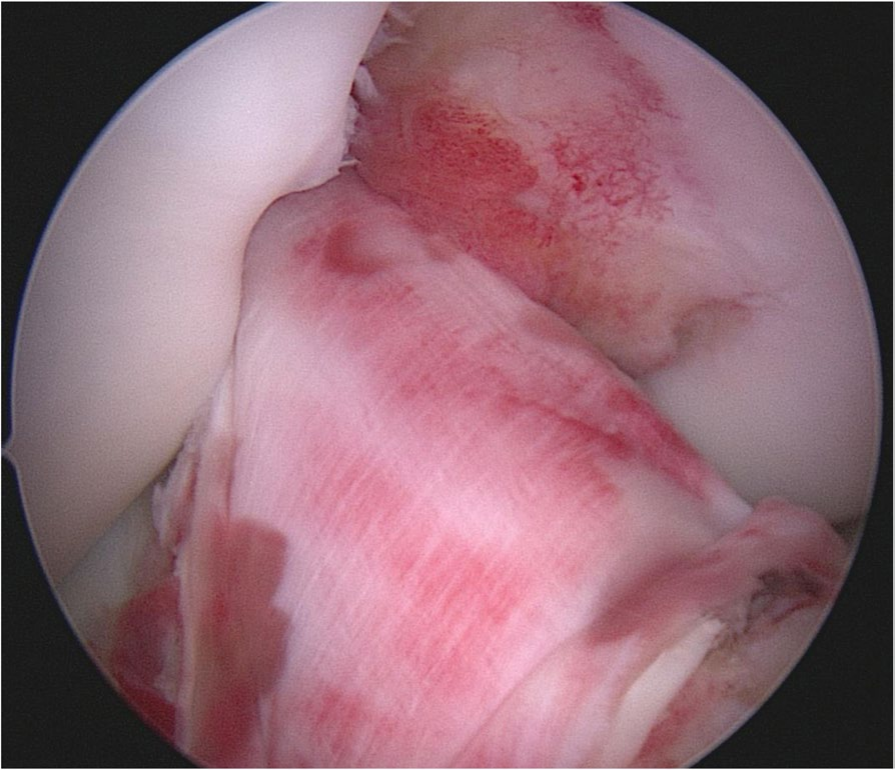
Arthroscopic picture of the collagen matrix ACL augmentation in a right knee

As shown in Figs. 1 and 2, a total of 102 cases fulfilled the inclusion criteria. Out of those, 15 cases were excluded due to the rupture location and 10 patients were lost to follow up. Thirty-one patients had a re-rupture and/or revision within 5 years. Of the remaining patients, 47 were available for clinical follow-up, 24 were available telephonically and answered our questionnaires without clinical examination. Patient demographics are shown in Table 1.

**Table 1:** Patient demographics

	Median (range)	Proportion	%
Age (years)	34 (20–65)		
Gender			
Female		41/102	40.2%
Male		61 /102	59.8%
Side			
Right		51/102	50%
Left		51/102	50%
Days until intervention	11 (0–21)		
Surgery			
DIS only		32/102	31.4%
DIS + APM		5/102	4.9%
DIS + Meniscus refixation		65/102	63.7%
Augmentation		45/102	44.1%
Only Collagen		35/102	34.3%
Only PRF		7/102	6.9%
Both		3/102	2.9%
Follow-up (months)	72 (60–117)		

*Abbreviations*: *DIS* Dynamic intraligamentary stabilization, *APM* Arthroscopic partial meniscectomy, *PRF* Platelet-rich fibrin

### Surgical technique

DIS Surgery was performed as previously described by Kohl et al. [18].

In most cases, there was a concomitant meniscal tear found. Based on individual need meniscal surgery was performed, as displayed in Table 1. DIS repair was augmented with a collagen fleece and/or PRF in 45/102 patients.

Two very similar fabrics of collagen matrices were used (Novocart, B. Braun Medical AG, Melsungen, Germany (*n* = 24) and Chondro-Gide, Geistlich Pharma AG, Wolhusen Switzerland (*n* = 14)). PRF was applied inside the Collagen sheathing in three cases. Single PRF was used in seven cases.

The following technique described formerly by Evangelopoulos et al. [8] was used for the Collagen Typ I/III membrane wrapping. In brief, the membrane was cut in oval shape and three PDS 3.0 sutures were placed (proximal, distal-medial, distal-lateral) at the edge of the collagen sheath. The prepared collagen was wrapped to the ruptured side at the anterior surface of the ACL. The proximal suture was tightened by a trans-osseous fixation along with the arming of the ACL. The distal sutures were shuttled through 2.4-mm k-wire holes at the anteromedial and anterolateral surfaces of the proximal tibia and tightened over the bony bridge between those holes. Figure 2 shows an arthroscopic picture of the augmentation technique.

PRP was extracted from patient blood samples as recommended for AngelTM System (Arthrex Inc., Naples, Florida, US). The combination with Arthrex activAT offers the opportunity to prepare autologous thrombin to process the PRP into PRF. The autologous PRF was applied inside the Collagen sheathing. In the seven cases no collagen coverage was used, it was applied directly to the rupture site.

Postoperatively, full weight bearing was allowed for the patients with isolated ACL ruptures and those with partial meniscectomy, using a brace for the first week. Due to meniscus refixation, partial weight bearing was requested in 65 cases for 6 weeks. After 2 weeks strength training and after 9 weeks sports training was started.

### Outcome measures

The minimum interval for final follow up was 60 months, median 72 (60–117) months. Patient reported outcome measures (PROMS) were collected for subjective assessment. All 102 patients answered Tegner, Lysholm and IKDC (subjective) questionnaires. Two groups were built, analogous to Ahmad et al. [1], one consisting of the patients with a Tegner activity level up to six and the other with Tegner 7 or more. This grouping showed a nearly balanced distribution of 40/102 versus 62/102. Furthermore, it characterizes the cutoff between recreational and competitive sports and a Tegner activity scale > 6 has been identified as a risk factor for failure [19].

In 47 patients available for physical examination the IKDC objective score (Group 1–4) was additionally determined. In these patients, joint stability was evaluated clinically and range of motion (ROM) was measured in degrees with a goniometer. Antero-posterior (a.p.) translation was examined at 20° flexion using the KT-1000 device, calculating the mean of three repetitive measures, respectively compared to the uninjured side.

### Endpoint definition of failure

Failure as endpoint was defined as traumatic re-rupture or conversion to ACL reconstruction within the follow-up interval. 

### Statistical analysis

SPSS statistics was used for data analysis (IBM SPSS Statistics, Version 25 for Windows). According to a Kolmogorov-Smirnov-Test data was not normally distributed and is therefore given as median with range. Simple descriptive statistics were used to answer the study questions. A Wilcoxon signed-rank test was used to compare ordinal data and a student’s t-test to compare quantitative data. The significance level was set at *p* < 0.05. Kaplan-Meier survival analysis and Log Rank test were performed, results are given in estimated survival and standard error (S.E.). Binominal logistic regression was used to identify factors influencing failure.

## Results

At a minimum 5-year follow-up 31/102 (30.4%) ACL repairs failed, resulting in a Kaplan-Meier estimated 5-year survival of 68.5% (standard error (S.E.) 4.8%). Survival rates showed no statistical difference whether augmentation (collagen fleece wrapping or PRF application or both of them) was applied or not (*p* = 0.812)..Survival was 68.4% without augmentation and 71.1% with augmentation.

Neither knee side nor gender had any impact on the outcome (*p* = 0.830, *p* = 0.131). Knee mobility and stability results are shown in Table 2. The table includes all patients with a clinically stable ACL and a full examination follow-up at minimum 5 years postoperatively.

**Table 2:** Results. Tegner activity scale ranges from 0 (minimal physical activity) to 10 (highest physical activity e.g. national elite soccer player) points. International Knee Documentation Commitee (IKDC) score ranges from 0 (maximum knee symptoms and disability) to 100 (no symptoms and full function) points. Lysholm Rating Scale is scored on a scale of 0 to 100, with higher scores indicating fewer symptoms and higher levels of functioning

	Median (range)	Proportion	%
Survival		71/102	69,6%
Non-augmented		39/57	68.4%
Augmented		32/45	71,1%
Pre-traumatic Tegner activity scale	7 (3–10)		
Group 1 (< 7)		40/102	39.2%
Group 2 (>/=7)		62/102	60.8%
Postoperative Tegner activity scale	6 (3–9)		
Group 1 (< 7)		40/71	56.3%
Group 2 (>/=7)		31/71	43.7%
IKDC	94.3 (49.4–100)		
Lysholm	94 (64–100)		
Active ROM	138° (120–155°)		
Passive ROM	145° (130–155°)		
Active ROM difference to uninjured side	0° (−8–10°)		
Passive ROM difference to uninjured side	0° (−10–20°)		
AP Translation difference to uninjured side	0 mm (−3-5 mm)		

None of the patients being available for follow-up had an extension deficit more than 0° neutral position or more than 5° compared to the uninjured knee. The median active ROM was 138° (120–155°) which was slightly lower than in the uninjured knee with median 140 ° (120–155°) (*p* = 0.008). Median passive ROM was 145° (130–155°), compared to 147° (130–160°) (*p* = 0.320). Median a.p. translation difference to the contralateral knee was 0 mm (− 3-5 mm). There was no statistical difference in the comparison of both side results (*p* = 0.056).

Functional outcome obtained by PROMS showed a median preinjury Tegner activity level of 7 (3–10) and a median postoperative Tegner of 6 (3–9) at final follow-up. Median Lysholm score was 94 (64–100) at final follow-up.

Of those patients with an intact ACL repair being available for physical examination, 45/47 (95.7%) had normal or nearly normal knee function based on the IKDC scoring system (A or B in every group), as displayed in Table 3; the median subjective IKDC score was 94.3 (49.4–100).

**Table 3:** IKDC objective outcome measures from knee examination after successful DIS

Group	Grade	Proportion	%
IKDC group 1 (effusion)	A	47/47	100%
IKDC group 2 (passive motion deficit)	A	38/47	80.9%
	B	9/47	19.1%
IKDC group 3 (ligament examination)	A	32/47	68.1%
	B	13/47	27.7%
	C	1/47	2.1%
	D	1/47	2.1%
IKDC group 4 (compartment findings)	A	37/47	78.7%
	B	9/47	19.1%
	D	1/47	2.1%

Grade A = normal; B = nearly normal; C = abnormal; D = severely abnormal

**Fig. 3:**
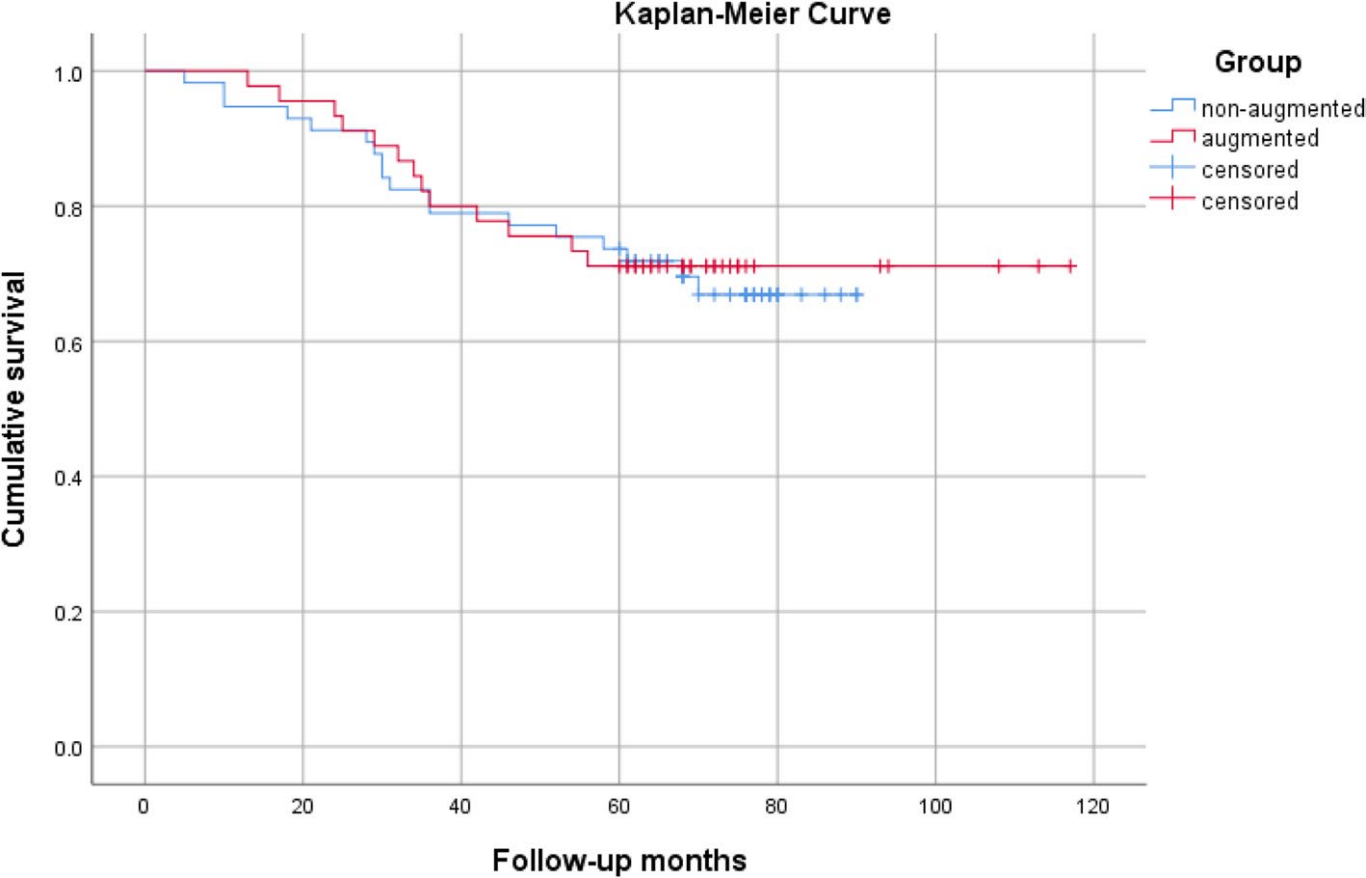
Kaplan-Meier survival curve of the augmented and non-augmented ACL repairs

**Fig. 4:**
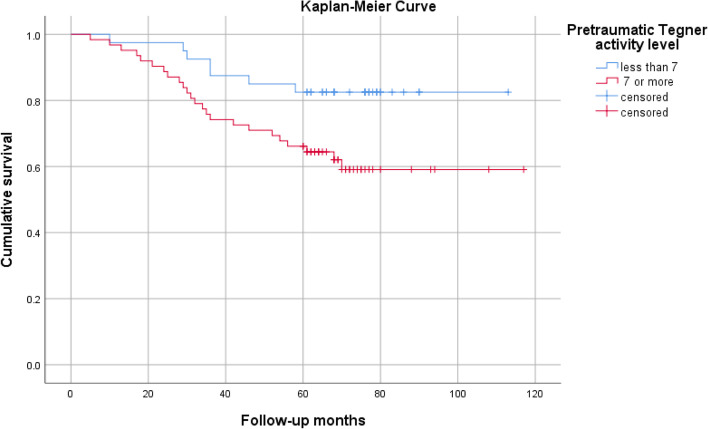
Kaplan-Meier survival curve of the two different pretraumatic Tegner activity level groups

One factor demonstrating direct influence on failure was a high pre-injury level of physical activity defined as a Tegner activity level of 7 or more. There was a significant higher failure rate in the more active group with Tegner activity level ≥ 7 with 38,7% compared to the group with a level < 7 with 17.5% (*p* = 0.022) using Log-Rank Test and Kaplan-Meier Analysis as shown in Figs. 3 and 4.

Another factor influencing success of the treatment was the patients` age as pointed out by binominal logistic regression (*p* = 0.016). The median age at surgery among the patients with intact DIS was 33 (20–65) years while it was 32 (21–49) years among the failures.

Unexpectedly, age and pretraumatic Tegner activity scale did not correlate with each other. In general, correlations between the predictor variables were low (*r* < 0.7), indicating that multi-collinearity was not a confounding factor in the analysis.

## Discussion

The most important finding of this study was that outcomes after DIS of proximal ACL ruptures could not be improved with additional augmentation. With a minimum follow-up of 5 years and over 100 patients, the current study is one of the first larger long-term analysis of DIS. Our results empower the findings of Ahmad et al. with an overall survival rate of 70% [1]. Patient selection seems to be a key factor in DIS repair. Risk factors for failure were a high pre-injury Tegner activity level (≥7) and younger age. Both was identified as a risk factor by Kösters et al. as well as by our research group, previously [19, 21]. Ahmad et al. demonstrated a significant drop of DIS survival from 70% to 56.4% if patients with a high pre-injury level of physical activity defined as Tegner activity level of ≥7 were selected [1]. Krismer et al. showed similar results with an increased failure rate (15,1% to 19%) in patients with high pre-injury Tegner score and mid-substance ACL tear [21].

Kösters et al. also reported that a Tegner activity level > 6 and age < 25 years were associated with higher failure rates in ACL reconstructions, indicating that high levels of activity and young age are not a specific risk factor for DIS failure but for failure of any surgical treatment of ACL ruptures [19].

The here presented augmentation techniques did not improve survival of DIS applied in proximal ACL ruptures. In contrast, Krismer et al. showed a decreased overall complication rate with augmentation. In the same publication, distal and mid-substance ruptures have been identified to be associated with a higher risk of failure if DIS is performed [21]. Because there is a higher risk of failure in distal and mid-substance ruptures, there might be a benefit for augmentation. We could not show this as we only included proximal ruptures in this study.

Patients not suffering failure of repair demonstrated good restoration of stability and high satisfaction. The subjective results are consistent with comparable studies of Henle et al. and Kösters et al., with Tegner, IKDC and Lysholm scores showing good or excellent functional results in more than 95% of those patients with intact ACL repair [13, 19].. None of our patients had a clinically relevant deficit in knee extension. This is in contrast to a prospective randomized clinical trial with 43 DIS, which showed an extension deficit of 5° or more in 7% of the cases after 2 years [19].

Within a short post-operative follow up period DIS showed comparable functional results and knee joint stability compared to ACLR in a controlled randomized study [25]. However, regarding mid- to long term results, the survival rates were markedly lower than those reported for established ACL reconstruction procedures [1, 19]. This was confirmed by our findings. Meta-analyses on ACL reconstructions reported failure rates between 3% and 7% [2, 9]. One reason for the high failure-rates seen with DIS repair may be a “windshield wiper” effect of the polyethylene thread causing friction and impairing healing at the femoral repair site if the tunnel position differs from the optimal position in the centre of the femoral ACL footprint.

This study has several limitations. Due to the retrospective character, selection bias in in performing DIS cannot be excluded. Regarding the main study question, the inhomogeneous augmentation technique also limits the generalizability. The number of ACL augmented only with PRF (*n* = 7) or both PRF and Collagen (*n* = 3) were too small for valid comparative statistical analysis. Combined with the retrospective character of the study this involves a risk for a type II error. Moreover, there was a heterogeneity in the rehabilitation protocol due to concomitant meniscal surgery. This could have been a confounding factor on the outcome. Twenty-four patients were not physically but telephonically available for follow-up. They answered the questionnaires but had no stability testing in physical examination. Therefore, some clinical instabilities may have been missed and survival rate may be overestimated by Kaplan-Meier analysis.

The focus of this study lies in the primary outcome regarding stability and functional outcome. The increase of osteoarthritis after DIS has not been examined so far, especially in comparison to conservative treatment and primary reconstruction. In our opinion this should be questioned in long-term research with follow-up over 20 years.

As a ligament- and tendon-sparing procedure, the advantage of the DIS technique is apparent and DIS repair of the ACL has its justification in the ACL decision tree. However, patient selection is crucial for this technique to be successful. DIS shows best results when used in proximal ruptures, less active and older patients. Augmentation with collagen matrix and PRF in the way it was performed in this study did not provide any benefit in these proximal ruptures.

## Conclusion

With a minimum follow-up of 5 years and over 100 patients, the current study is one of the first larger long-term outcome reports of DIS. The overall survival rate of DIS in this case-series was 69.6%. Collagen fleece wrapping and local PRF application did not show statistical differences regarding survival rates. The factors increasing the risk of failure were a younger age and a pre-injury Tegner activity level of ≥7. Patients not suffering failure of repair demonstrated high satisfaction. Nevertheless, the results are inferior to those of established ACL reconstruction procedures.

## Data Availability

All data analysed during this study are included in this published article.

## Abbreviations

a.p.: Anterior-posterior
APM: Arthroscopic meniscectomy
ACL: Anterior cruciate ligament
ACLR: Anterior cruciate ligament reconstruction
DIS: Dynamic intraligamentary stabilization
FU: Follow-up
IKDC: International Knee Documentation Commitee
PDGF-AB: Platelet-derived growth factor-AB
PRF: Platelet-rich fibrin
PROMS: Patient reported outcome measures
PRP: Platelet-rich plasma
TGF**-**β1: Transforming growth factor**-**β1 S.E.: standard error

## References

[1] Ahmad SS, Schurholz K, Liechti EF, Hirschmann MT, Kohl S, Klenke FM (2020). Seventy percent long-term survival of the repaired ACL after dynamic intraligamentary stabilization. Knee Surg Sports Traumatol Arthrosc.

[2] Andernord D, Desai N, Björnsson H, Ylander M, Karlsson J, Samuelsson K (2015). Patient predictors of early revision surgery after anterior cruciate ligament reconstruction: a cohort study of 16,930 patients with 2-year follow-up. Am J Sports Med.

[3] Ateschrang A, Schreiner AJ, Ahmad SS, Schroter S, Hirschmann MT, Korner D (2019). Improved results of ACL primary repair in one-part tears with intact synovial coverage. Knee Surg Sports Traumatol Arthrosc.

[4] Davarinos N, O'Neill BJ, Curtin W (2014). A brief history of anterior cruciate ligament reconstruction. Advanc Orthop Surg.

[5] Dohan Ehrenfest DM, de Peppo GM, Doglioli P, Sammartino G (2009). Slow release of growth factors and thrombospondin-1 in Choukroun's platelet-rich fibrin (PRF): a gold standard to achieve for all surgical platelet concentrates technologies. Growth Factors.

[6] Eggli S, Kohlhof H, Zumstein M, Henle P, Hartel M, Evangelopoulos DS (2015). Dynamic intraligamentary stabilization: novel technique for preserving the ruptured ACL. Knee Surg Sports Traumatol Arthrosc.

[7] Eggli S, Roder C, Perler G, Henle P (2016). Five year results of the first ten ACL patients treated with dynamic intraligamentary stabilisation. BMC Musculoskelet Disord.

[8] Evangelopoulos DS, Kohl S, Schwienbacher S, Gantenbein B, Exadaktylos A, Ahmad SS (2017). Collagen application reduces complication rates of mid-substance ACL tears treated with dynamic intraligamentary stabilization. Knee Surg Sports Traumatol Arthrosc.

[9] Fältström A, Hägglund M, Magnusson H, Forssblad M, Kvist J (2016). Predictors for additional anterior cruciate ligament reconstruction: data from the Swedish national ACL register. Knee Surg Sports Traumatol Arthrosc.

[10] Gagliardi AG, Carry PM, Parikh HB, Traver JL, Howell DR, Albright JC (2019). ACL repair with suture ligament augmentation is associated with a high failure rate among adolescent patients. Am J Sports Med.

[11] Hardy A, Casabianca L, Andrieu K, Baverel L, Noailles T (2017). Complications following harvesting of patellar tendon or hamstring tendon grafts for anterior cruciate ligament reconstruction: systematic review of literature. Orthop Traumatol Surg Res.

[12] He L, Lin Y, Hu X, Zhang Y, Wu H (2009). A comparative study of platelet-rich fibrin (PRF) and platelet-rich plasma (PRP) on the effect of proliferation and differentiation of rat osteoblasts in vitro. Oral Surg Oral Med Oral Pathol Oral Radiol Endod.

[13] Henle P, Röder C, Perler G, Heitkemper S, Eggli S (2015). Dynamic Intraligamentary stabilization (DIS) for treatment of acute anterior cruciate ligament ruptures: case series experience of the first three years. BMC Musculoskelet Disord.

[14] Jacobi M, Reischl N, Ronn K, Magnusson RA, Gautier E, Jakob RP (2016). Healing of the acutely injured anterior cruciate ligament: functional treatment with the ACL-Jack, a dynamic posterior drawer brace. Adv Orthop.

[15] Kaur M, Ribeiro DC, Theis J-C, Webster KE, Sole G (2016). Movement patterns of the knee during gait following ACL reconstruction: a systematic review and Meta-analysis. Sports Med.

[16] Kessler MA, Behrend H, Henz S, Stutz G, Rukavina A, Kuster MS (2008). Function, osteoarthritis and activity after ACL-rupture: 11 years follow-up results of conservative versus reconstructive treatment. Knee Surg Sports Traumatol Arthrosc.

[17] Kohl S, Evangelopoulos DS, Ahmad SS, Kohlhof H, Herrmann G, Bonel H (2014). A novel technique, dynamic intraligamentary stabilization creates optimal conditions for primary ACL healing: a preliminary biomechanical study. Knee.

[18] Kohl S, Evangelopoulos DS, Kohlhof H, Hartel M, Bonel H, Henle P (2013). Anterior crucial ligament rupture: self-healing through dynamic intraligamentary stabilization technique. Knee Surg Sports Traumatol Arthrosc.

[19] Kösters C, Glasbrenner J, Spickermann L, Kittl C, Domnick C, Herbort M (2020). Repair with dynamic Intraligamentary stabilization versus primary reconstruction of acute anterior cruciate ligament tears: 2-year results from a prospective randomized study. Am J Sports Med.

[20] Krismer AM, Cabra RS, May RD, Frauchiger DA, Kohl S, Ahmad SS (2017). Biologic response of human anterior cruciate ligamentocytes on collagen-patches to platelet-rich plasma formulations with and without leucocytes. J Orthop Res.

[21] Krismer AM, Gousopoulos L, Kohl S, Ateschrang A, Kohlhof H, Ahmad SS (2017). Factors influencing the success of anterior cruciate ligament repair with dynamic intraligamentary stabilisation. Knee Surg Sports Traumatol Arthrosc.

[22] Krogsgaard MR, Dyhre-Poulsen P, Fischer-Rasmussen T (2002). Cruciate ligament reflexes. J Electromyogr Kinesiol.

[23] Meister M, Koch J, Amsler F, Arnold MP, Hirschmann MT (2018). ACL suturing using dynamic intraligamentary stabilisation showing good clinical outcome but a high reoperation rate: a retrospective independent study. Knee Surg Sports Traumatol Arthrosc.

[24] Murray MM (2009). Current status and potential of primary ACL repair. Clin Sports Med.

[25] Schliemann B, Glasbrenner J, Rosenbaum D, Lammers K, Herbort M, Domnick C (2018). Changes in gait pattern and early functional results after ACL repair are comparable to those of ACL reconstruction. Knee Surg Sports Traumatol Arthrosc.

[26] Shea KG, Carey JL, Richmond J, Sandmeier R, Pitts RT, Polousky JD (2015). Management of anterior cruciate ligament injuries. J Bone Joint Surg Am.

[27] Strehl A, Eggli S (2007). The value of conservative treatment in ruptures of the anterior cruciate ligament (ACL). J Trauma.

[28] Vavken P, Murray MM (2011). The potential for primary repair of the ACL. Sports Med Arthrosc Rev.

[29] von Porat A, Roos EM, Roos H (2004). High prevalence of osteoarthritis 14 years after an anterior cruciate ligament tear in male soccer players: a study of radiographic and patient relevant outcomes. Ann Rheum Dis.

[30] Zimny ML, Schutte M, Dabezies E (1986). Mechanoreceptors in the human anterior cruciate ligament. Anat Rec.

